# Enhanced testing can substantially improve defence against several types of respiratory virus pandemic

**DOI:** 10.1016/j.epidem.2024.100812

**Published:** 2025-01-13

**Authors:** James Petrie, James A. Hay, Oraya Srimokla, Jasmina Panovska-Griffiths, Charles Whittaker, Joanna Masel

**Affiliations:** 1 Pandemic Sciences Institute, University of Oxford; 2 Big Data Institute, University of Oxford; 3 Nuffield Department of Medicine, University of Oxford; 4 UK Health Security Agency; 5 The Queen’s College, University of Oxford; 6 Department of Infectious Disease Epidemiology, Imperial College London; 7 Ecology & Evolutionary Biology, University of Arizona

## Abstract

Mass testing to identify and isolate infected individuals is a promising approach for reducing harm from the next acute respiratory virus pandemic. It offers the prospect of averting hospitalizations and deaths whilst avoiding the need for indiscriminate social distancing measures. To understand scenarios where mass testing might or might not be a viable intervention, here we modelled how effectiveness depends both on characteristics of the pathogen (*R*_0_, time to peak viral load) and on the testing strategy (limit of detection, testing frequency, test turnaround time, adherence). We base time-dependent test sensitivity and time-dependent infectiousness on an underlying viral load trajectory model. We show that given moderately high public adherence, frequent testing can prevent as many transmissions as more costly interventions such as school or business closures. With very high adherence and fast, frequent, and sensitive testing, we show that most respiratory virus pandemics could be controlled with mass testing alone.

## 1 Introduction

Respiratory virus pandemics pose a major threat to human health and well-being, as evidenced by the impacts of COVID-19 and 1918 influenza pandemics. The UK government estimates between 5-25% chance of a new pandemic the magnitude of COVID-19 occurring within the next 5 years, which could potentially lead to up to 800,000 deaths as well as extensive social distancing [1]. Given the massive potential harm of future pandemics, it is important to make disease control tools more reliable. Vaccines and therapeutics currently take months or years to design, test, and manufacture, and are not guaranteed to work. Social distancing imposes a huge burden on the population and is not sustainable for a long period. With low enough disease prevalence, contact tracing can prevent transmissions more efficiently than social distancing [2]. However, contact tracing is often not reliable enough, on its own, to control airborne pathogens, especially for the short latent periods combined with pre-symptomatic and/or asymptomatic transmission seen for SARS-CoV-2 [3, 4]. Before there is another major pandemic, there is time to prepare strategies, technology, and infrastructure. It is important to understand which approaches would be most useful to invest in.

Mass testing, defined as frequent testing of most of the population and isolation of positives, was proposed as a promising approach to contain SARS-CoV-2 [5, 6, 7, 8, 9, 10, 11, 12]. On a relatively small scale (tens of thousands of people), several universities, professional sports teams and film studios succeeded in operating in-person with low disease burden during the COVID-19 pandemic by frequently testing people [13, 14, 15, 16]. Children in secondary school in the UK were recommended to test twice weekly in March 2021 [17], and Slovakia performed two rounds of mass antigen testing, which Pavelka et al [18] estimated caused a 70% reduction in prevalence. However, prior to the availability of vaccines, and even with the pooling of samples, there was not enough testing capacity outside of China [19] to consistently test more than 0.1% of the nation-wide population per day [20].

Mass testing of an entire population would be logistically complicated, and expensive, raising the question of whether scaling it up would be worth it, even in the light of the past success of smaller scale implementations. It would also require public adherence to testing and isolation, and the capacity to do a large number of tests early in a pandemic. While scaling up is difficult to do in real time during a pandemic, sufficient preparation might mitigate current limitations to mass testing. Appendix A discusses how the development of technology and infrastructure might optimize for scalability, cost effectiveness, and speed.

Our primary aim is to provide a framework for understanding the potential effectiveness of different mass testing strategies (similar to Fraser et al.’s 2004 work on contact tracing [4]). If mass testing is likely to be effective, then this effectiveness should be weighed against expected costs to inform investments into technology and infrastructure intended to increase capacity beyond that available for SARS-CoV-2. We consider the effectiveness of mass testing given a range of likely values for viral characteristics (*R*_0_ and generation time), as well as for testing policy characteristics (limit of detection, test delay, test frequency, and population adherence). Similar to previous studies [21, 22, 23], we evaluate the potential impact of mass testing on reducing transmission, by modeling different mass testing strategies with time-dependent test sensitivity conditional on viral load trajectories. Like Middleton and Larremore [24], we treat infectiousness as a function of the same underlying viral load trajectories. In contrast to previous work, our aim is to determine which pandemic scenarios mass testing would be useful for, rather than whether it would work specifically for SARS-CoV-2. Our model is available as an interactive application (https://frequent-testing.shinyapps.io/shinyapp), to enable easy exploration of different mass testing policies and modelling assumptions.

## 2 Methods

We start by computing the expected number of transmissions from a person who tests regularly and isolates perfectly when they learn they are positive. We later adjust this value to account for imperfect adherence, and then estimate the population-level effect of mass testing under the assumption that the population is well-mixed. Default parameter values are chosen based on SARS-CoV-2, and then we explore different parameter values to represent other pathogens. The model parameters defined in this section are summarized in Table 1.

### 2.1 Viral Load Trajectory

**Figure 1:**
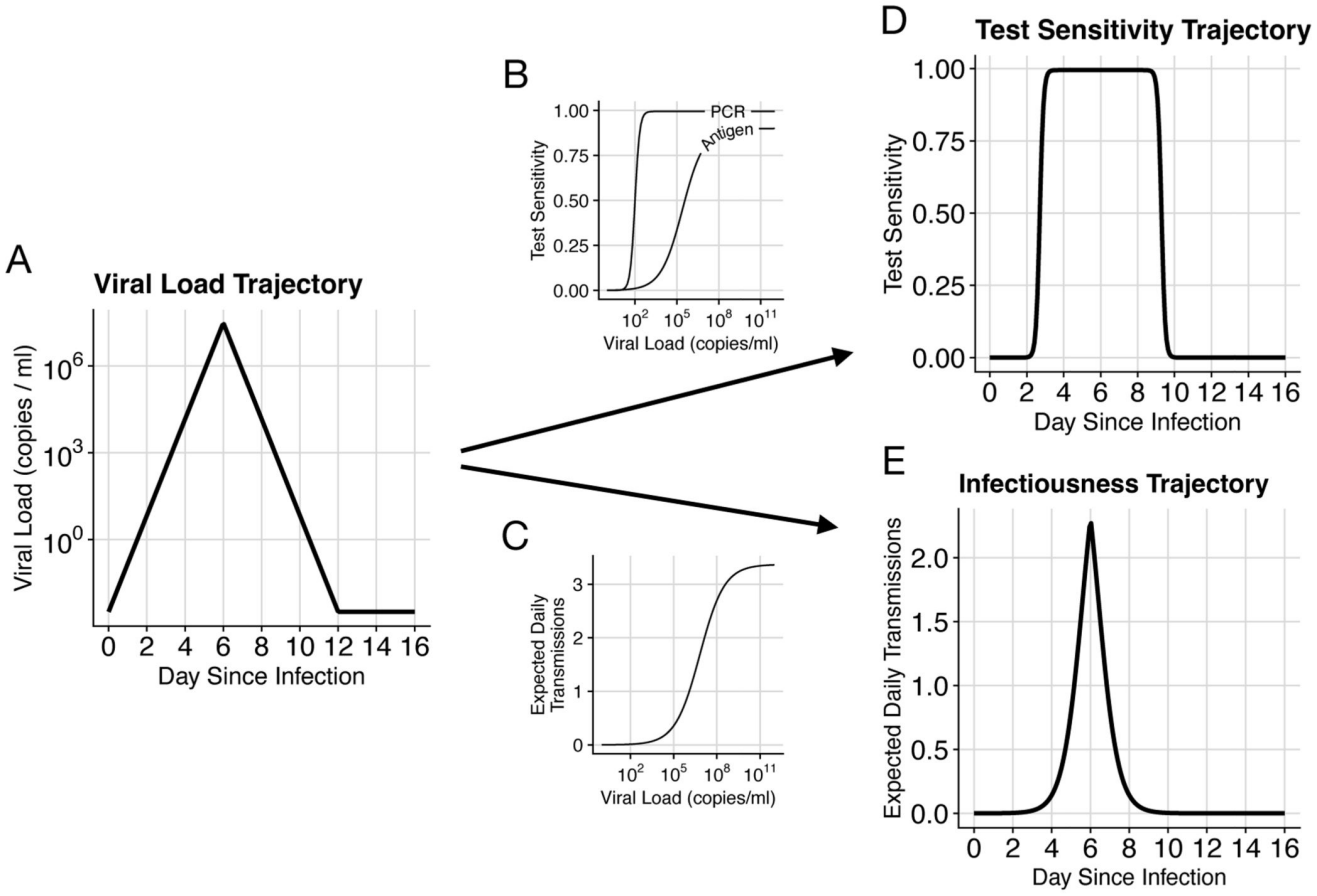
Infectiousness and test sensitivity over time are estimated based on simulated viral load trajectories. (A) An example viral load trajectory, characterized by peak viral load and times from infection to peak, and from peak to recovery. (B) Test sensitivity vs. viral load for typical PCR and antigen tests. (C) Expected transmissions per day vs. viral load. (D) Test sensitivity vs. time since infection for this pathogen, computed by passing the time series in (A) through function (B). (E) Expected daily transmissions vs. time since infection, computed by passing the time series in (A) through function (C). The area under the curve in (E) is *R*_0_ for this pathogen.

Trajectories of log viral load for acute respiratory infections like influenza or SARS-CoV-2 are well modelled by piece-wise linear curves [15, 25, 22] (Figure 1A). For simplicity, we neglect within-population diversity in trajectories; in Appendix B we demonstrate that this assumption does not substantially change the predicted effectiveness of PCR testing against most pathogens (due to approximate linearity in relevant parts of the parameter space shown in Figure 6). This allows us to characterize pathogens according to the mean time from infection to reach peak viral load *τ*_*p*_, the mean time from peak viral load to recovery, *τ*_*r*_, and the peak viral load, *V*_*p*_. The initial viral load when infected, *V*_0_, is set to 3 · 10^−3^ based on initial infection with one viral particle and roughly 300ml of respiratory fluid [26]. Log viral load *V* is then:

(1) $${\text{log}}_{10}(V(t))=\{\begin{array}{cc} \log_{10}\left(V_{0}\right)\cdot \left(1-\frac{t}{\tau_{p}}\right)+{\text{log}}_{10}\left(V_{p}\right)\cdot \frac{t}{\tau_{p}}, & \text{for}\;0<t\leq \tau_{p}\\ \log_{10}\left(V_{p}\right)\cdot \left(1-\frac{t-\tau_{p}}{\tau_{r}}\right)+{\text{log}}_{10}\left(V_{0}\right)\cdot \frac{t-\tau_{p}}{\tau_{r}}, & \text{for}\;\tau_{p}\leq t<\tau_{p}+\tau_{r}\\ -\infty , & \text{otherwise}\; \end{array}$$

For most of this analysis, we assume viral load trajectories are symmetric, i.e. *τ*_*p*_ = *τ*_*r*_; alternative trajectory shapes are analyzed in Appendix D and can be explored further in the interactive app at https://frequent-testing.shinyapps.io/shinyapp. This assumption of symmetric trajectories slightly underestimates the effectiveness of mass testing compared to right skewed trajectories (*τ*_*r*_ *> τ*_*p*_), where transmissions occur later in infection and are therefore easier to prevent

Note that inactive virus material can persist for a long time following clearance of replication competent virions, which can prolong the time from peak viral load to a negative test result. This process might therefore be better modeled as the sum of exponential declines rather than a single exponential decline. In this context, our piecewise curves can be interpreted as replication-competent viral load peaking at *V*_*P*_ . Test sensitivity (probability that a sample from an infected person is classified as positive) depends strongly on the person’s viral load at the time the sample is collected [27]. We neglect the contribution to test sensitivity from non-replication competent viral material, because its only effect is to catch infected individuals too late to prevent transmission, in particular when testing is less frequent.

### 2.2 Test Sensitivity Depending on Viral Load

We model the dependence of test sensitivity, *S*, on viral load, *v*, with the sigmoid function:

(2) $$S\left(v;S_{max},k,LOD_{50}\right)=S_{max}\frac{1}{1+e^{-k\left({\text{log}}_{10}(v)-{\text{log}}_{10}\left({\text{LOD}}_{50}\right)\right)}}$$

This equation is characterized by peak test sensitivity, *S*_*max*_, the viral load LOD_50_ at which *S*_*max*_*/*2 of samples from infected people are classified as positive, and a parameter *k* that specifies the width of the intermediate region. For PCR tests, we set LOD_50_ = 10^2^ copies/ml and *k* = 6 so that the distance between 5% and 95% sensitivity is about a multiple of 10 as in [27]. We set *S*_*max*_ = 99.5% to account for sample mishandling. Antigen tests are modeled as having a limit of detection of 10^5.4^ copies/ml and *k* = 1.3 based on the average sensitivity that Wagenhauser et al. measured for the ancestral SARS-CoV-2 variant [30] (although this varies considerably between manufacturers, between variants of the same pathogen, and presumably between pathogens). The maximum sensitivity, *S*_*max*_, is reduced to 90% to account for errors in self-administered antigen tests. Default PCR and antigen sensitivity curves are shown in Figure 1B.

### 2.3 Expected Transmission Rate Depending on Viral Load

While there is consensus that higher viral load increases the expected number of transmissions per day, *T* (*v*), quantitative data on this relationship are limited. Ke et al. [29] used the measured relationship between SARS-CoV-2 viral load (as assessed by PCR) and cell culture positivity as a proxy for the relationship between viral load and transmission. We use Ke et al.’s saturation model [29] in Equation 3, with SARS-CoV-2-inspired default values of *N*_*C*_ = 13 contacts per day [28], shape parameter *h* = 0.51, and an infectiousness midpoint of *K*_*m*_ = 8.9 · 10^6^copies/ml (see Appendix E for a sensitivity analysis of *K*_*m*_). Ke et al. included a parameter *θ* to reduce the maximum infectiousness below 100%; we increased this from 0.2 to 0.3, corresponding to an increase from 18% to 26% per contact.

(3) $$T\left(v;N_{C},h,K_{m},\theta \right)=N_{C}\cdot \left(1-e^{-\theta \frac{v^{h}}{v^{h}+K_{m}^{h}}}\right)$$

These values of *K*_*m*_ and *θ* are in broad agreement with Marc et al.’s [31] use of SARS-CoV-2 contact tracing data to connect estimated viral load at time of exposure with transmission probability. Our assessment of agreement is focused on non-household contacts, because household contacts typically are exposed over multiple days, corresponding to multiple opportunities for transmission, with a correspondingly higher overall maximum probability of infection. Equation 3 for expected daily transmissions as a function of viral load is shown in Figure 1C.

### 2.4 Test Sensitivity and Expected Transmissions over Time

Test sensitivity over time is computed as the composition of Equations 1 and 2, *S*(*V* (*t*)), shown in Figure 1D. Similarly, the expected rate of transmissions as a function of time since infection is computed as the composition of Equations 1 and 3, *T*(*V* (*t*)), shown in Figure 1E. For pathogens where noticeable symptoms cause people to reduce interactions and therefore transmit to fewer people, *N*_*C*_ can be made a function of time since infection, as explored in Appendix C (Figures 7 and 8).

### 2.5 Default Expected Transmissions per Infection (*R*_0_)

The expected number of transmissions per infected person (in an immuno-naive population with no behaviour modifications) is $R_{0}=\int_{0}^{\infty }T(V(t))\cdot dt$. I.e., total (expected) transmission is equal to the integral of (expected) transmission over time. To achieve a given *R*_0_ value with *τ*_*p*_ fixed, we modify the peak viral load, *V*_*p*_, according to the relationship of Equation 1.

### 2.6 Detection Probability over Time

Consider a testing policy with a set period, *ρ*, between sequential tests. An individual whose first post-infection sample is collected *κ* days after infection (we refer to this as the offset) will have a negative status on day *t* following infection (i.e. will have tested negative on all samples collected on or before day *t*, if any) with probability

(4) $$ProbAllNegative(t;\kappa ,\rho )=\{\begin{array}{c} 1,\;\;\;\;\;\text{if}\;t<\kappa \\ {\text{Π}}_{i=0}^{\left\lfloor (t-\kappa )/\rho \right\rfloor }(1-S(V(i*\rho +\kappa ))),\;\;\text{otherwise}\; \end{array}$$

We compute the probability that a person knows they are positive at time *t* after infection by averaging across offset times, *κ ∼ Uniform*(0, *ρ*) (because samples are collected every *ρ* days and infection timing is independent of test timing). Allowing for a fixed delay time, *δ*, between sample collection and receiving results, the probability that at least one sample taken before *t* − *δ* is detected as positive is

(5) $$\begin{array}{l} ProbAnyPositive(t;\delta ,\rho )=E_{\kappa }[1-ProbAllNegative(t-\delta ;\kappa ,\rho )]\\ \;\;\;\;=\frac{1}{\rho }\int_{0}^{\rho }[1-ProbAllNegative(t-\delta ;\kappa ,\rho )]d\kappa \end{array}$$

### 2.7 Transmissions Prevented by Isolation

When informing a person that they are infectious causes them to isolate effectively, the effective transmission rate at time *t* is reduced by a factor *ProbAnyPositive*(*t*). The expected number of transmissions prevented by frequent testing followed by perfect isolation after testing positive is then given by the integral of *ProbAnyPositive*(*t*) · *T* (*V* (*t*)) over time. We define *σ* as the fraction of transmissions that would be prevented by frequent testing given perfect adherence.

(6) $$\sigma (\delta ,\rho )=\frac{\int_{0}^{\infty }[ProbAnyPositive(t;\delta ,\rho )\cdot T(V(t))]dt}{R_{0}}$$

### 2.8 Transmissions with Partially Adhered to Testing and Isolation (*R_e_*)

Generalizing to imperfect adherence, let *γ* be the probability that a person adheres to regular testing, and *β* capture the degree to which a positive test results causes a person to reduce their transmission, with a fraction *σ* of their counterfactual expected transmissions having not yet occurred. Then assuming a well mixed population, mass testing reduces the effective reproduction number according to:

(7) $$R_{e}(\gamma ,\beta ,\delta ,\rho )=R_{0}\cdot (1-\gamma \cdot \beta \cdot \sigma (\delta ,\rho ))$$

## 3 Results

**Figure 2:**
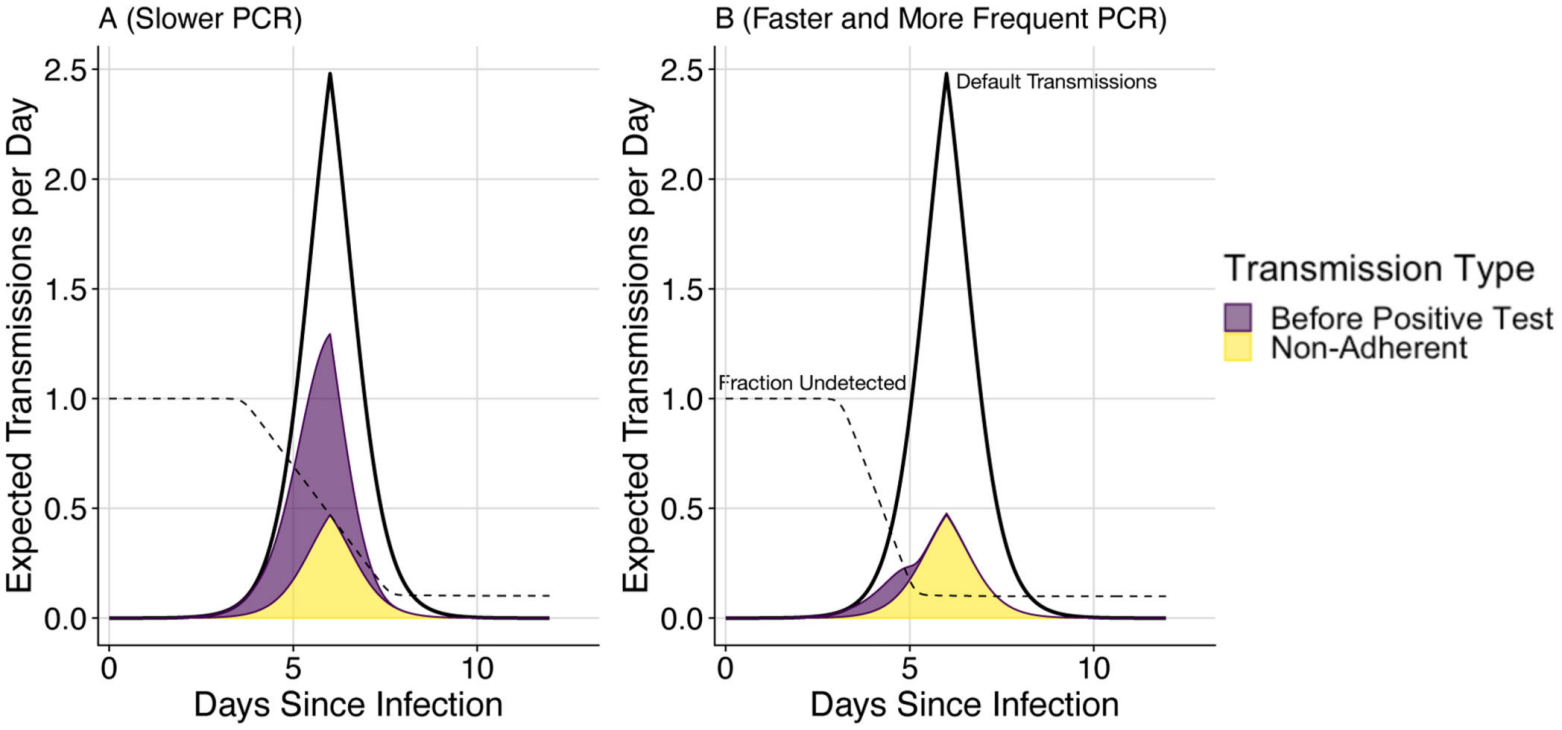
Expected transmissions under different testing strategies. The upper black lines illustrate the baseline scenario (with *R*_0_ = 4.0 expected transmissions). With mass testing, the yellow areas show transmissions occurring from people who don’t adhere to either testing or isolation, and the purple areas show transmissions from people that do adhere to testing and would isolate, but had not yet received a positive test result. The purple and yellow areas are stacked and do not overlap. Dashed lines show the fraction of infected people who have not yet tested positive. A) Testing every 4 days with a 24 hour test turnaround. B) Testing every 2 days with a 12 hour test turnaround. With less frequent and more delayed tests (A), transmission reduction is modest. With more frequent and less delayed tests (B), most transmissions are prevented, and the remaining transmissions are either early in infection or due to non-adherence to the testing and isolation policy. Both scenarios use tests with a limit of detection of 10^2^ copies/ml (as described by Equation 2), 90% adherence to testing, 90% effective isolation (conditional on being a person who gets tested), 6 days from infection to peak viral load, and 6 days from peak viral load to recovery.

Our results show that high adherence combined with frequent, rapid mass testing provide an effective strategy to prevent infections and hospitalizations. To better understand the effectiveness of mass testing, we decompose transmissions over the course of a typical infection into two broad failure modes: (1) non-adherence or (2) insufficiently sensitive, frequent, or rapid testing. Figure 2 shows expected transmissions over time when using two testing strategies against an example pathogen. Individuals that do not adhere to testing or do not effectively isolate when detected as positive, transmit on the same schedule as in the unmitigated scenario, as shown by the yellow regions in Figure 2 (the yellow regions in A and B are identical because adherence is the same for both strategies). If testing is sufficiently frequent and results are not substantially delayed, then individuals adhering to the policy transmit at a much lower rate. In this case, transmissions from adherent individuals are usually earlier in infection, as shown by the purple region in Figure 2B. When testing is more delayed (in this example 24 hours instead of 12 hours) and less frequent (here every 4 days instead of every 2 days), then adherent individuals transmit substantially more and relatively later in infection, as shown by the larger purple region in Figure 2A.

**Figure 3:**
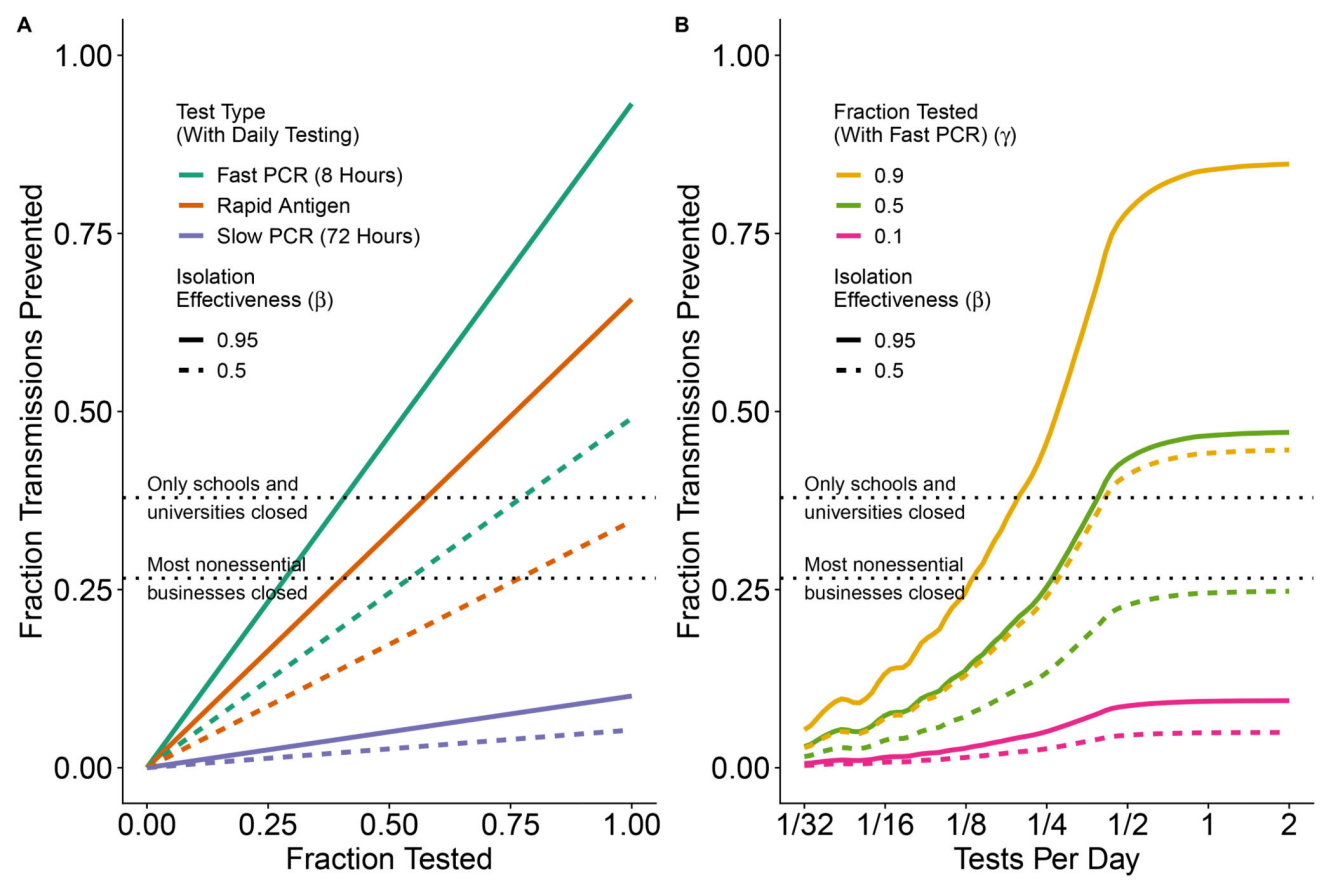
Fraction of (ancestral variant) SARS-CoV-2 transmissions prevented depends on adherence to testing and isolation (A) and test frequency (B). SARS-CoV-2 is parameterized as taking 5 days to reach peak viral load, symptoms occurring on the same day as peak viral load, a 50% reduction in transmissions after symptoms, and *R*_0_ = 2.75 (including the behaviour modification from symptoms). In (A) we model daily testing with either a rapid antigen test (0 hour delay, *LOD*_50_ = 10^5.4^, orange), or a fast PCR test (8 hour delay, *LOD*_50_ = 10^2^, green), or a slow PCR test (72 hour delay, *LOD*_50_ = 10^2^, blue). For (B) we model only the fast PCR test. For this pathogen, the benefit of frequent testing saturates when testing more often than every 2 days (B). Estimates by Brauner et al. [32] of the fraction reduction in transmissions from school and university closures (38%) and business closures (27%) are added for comparison (dashed lines).

Figure 3 compares mass testing to other commonly used strategies against the ancestral variant of SARS-CoV-2, expressing results as transmitted averted (reduction in *R*_*e*_) as a function of adherence to testing, effectiveness of isolation, the frequency of testing, and the type of test. The proportion of transmissions prevented depends linearly on adherence to testing and isolation (Figure 3A), as described by the dependence on *γ* and *β* in Equation 7. The benefit from frequent testing saturates when testing more than every 2 days for the ancestral variant of SARS-CoV-2 (Figure 3B), with 70% more infections averted when testing every 2 days compared to every 4 days. Fast PCR testing every 3 days with 50% adherence to testing and 95% effective isolation (Figure 3B) achieves the same fraction reduction in transmissions as school and university closures (38%, as assessed by Brauner et al. [32]). Higher adherence with more frequent testing is able to further reduce transmissions (curves in figure 3B above upper dashed line).

**Figure 4:**
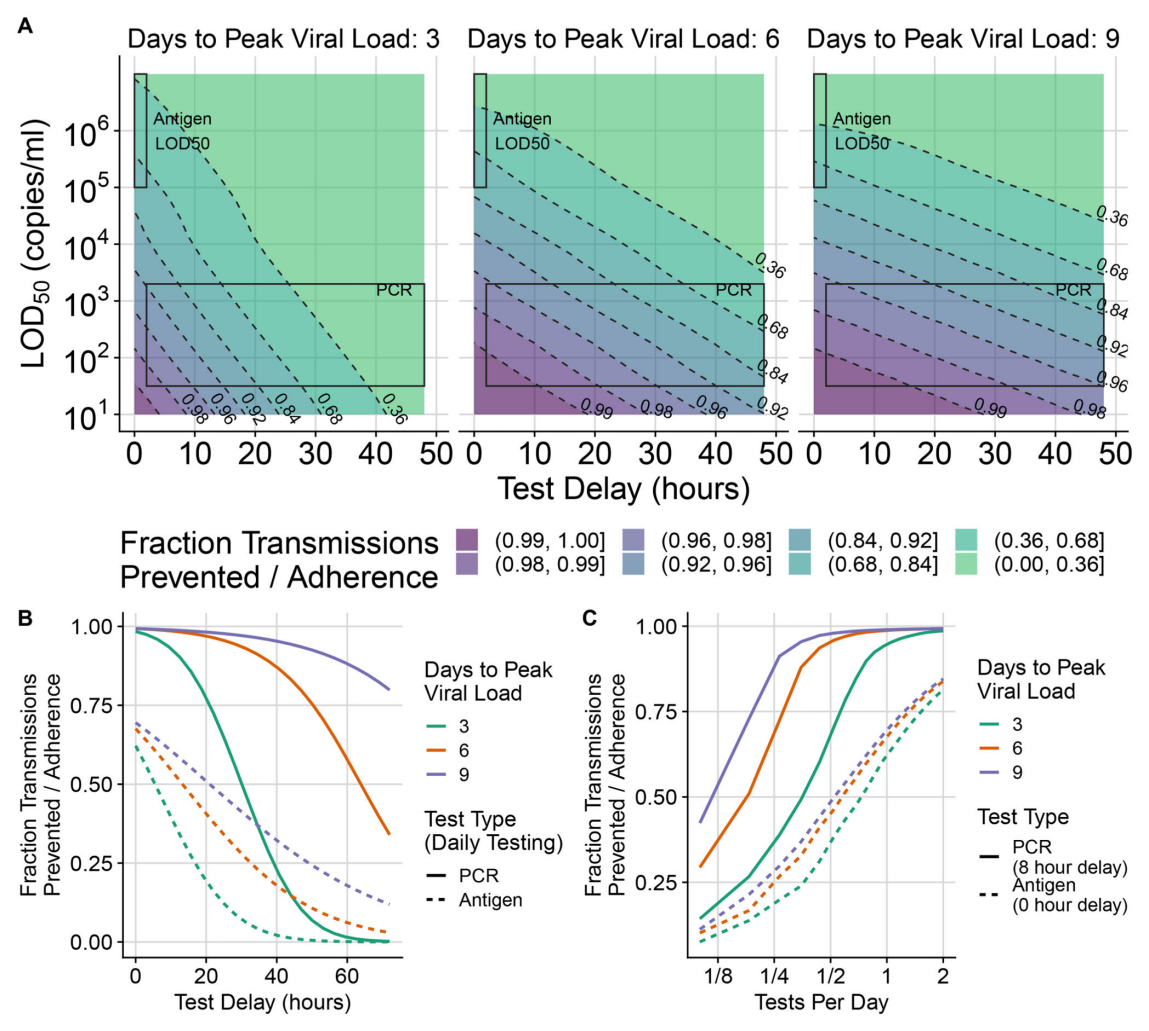
The fraction of transmissions prevented by mass testing depends on test frequency, *LOD*_50_, and test delay for three hypothetical pathogens that take 3, 6, and 9 days to reach peak viral load. The extent of transmission reduction from an infected person who tests daily and isolates effectively if positive depends on test turnaround time (x-axis of (A)), test limit of detection (y-axis of (A)), and time to peak viral load, with peak load varying accordingly to maintain *R*_0_ = 3 (3 panels in (A)). For shorter latent periods (left of (A)) delays are more important (closer contour lines along horizontal transect). Test sensitivity is similarly important for all latent periods; (similarly spaced contour lines along vertical transect). Shaded areas indicate likely *LOD*_50_ and delay time values for PCR vs. antigen tests. We use *k* = 6 and *S*_*max*_ = 99.5% from Equation 2 for all tests in (A) and for PCR tests in (B) and (C). This is overoptimistic about the maximum sensitivity (*S*_*max*_) of antigen tests, and invokes a sharper transition region than typical, so the shaded region in the upper left corner of each subplot in (A) corresponds with the *LOD*_50_ of antigen tests but not the shape of their test sensitivity curve. For (B) and (C), antigen tests are computed more accurately with *LOD*_50_ = 10^5.4^, *k* = 1.3, and *S*_*max*_ = 0.9. Rapid PCR tests (e.g. 8 hour turnaround) achieve dramatic transmission reduction under all conditions considered. Calculations assume perfect adherence to daily testing and to isolation following a positive test result. The fractional reduction in transmissions is proportional to adherence, so results can be modified for other scenarios by multiplication (e.g. if the fraction reduction is 0.8 with perfect adherence, it would be 0.4 if 50% of people tested frequently and isolated effectively if positive). Pooled PCR tests will be shifted upwards by the appropriate pooling factor, and are likely to also have increased delay. (B) shows cross-sections that hold the limit of detection constant while varying the test delay and (C) shows intervals other than daily testing.

Fast reporting of test results is more important for diseases that reach peak viral load quickly (e.g. influenza). In Figure 4A, the fraction reduction in transmissions for a perfectly adhering individual is shown as a function of *LOD*_50_ and test delay for three example pathogens that take 3, 6, and 9 days to reach peak viral load, with peak viral load modified so that *R*_0_ = 3 for all pathogens. The contours are much steeper for the pathogen that takes 3 days to reach peak viral load than for the other pathogens, which means that the relative importance of fast reporting is greater. When using PCR tests, the number of transmissions with a 20-hour delay compared to a 10-hour delay is 3.2x larger with 3 days to peak viral load, 2.0x larger with 6 days to peak viral load, and 1.6x larger with 9 days to peak viral load. With a low limit of detection (10^2^ copies/ml) and fast reporting (10 hours), adhered-to daily testing is able to prevent more than 92% of transmissions for a pathogen with 3 days to peak viral load, and almost 99% of transmissions for a pathogen with 9 days to peak viral load.

Because of antigen tests’ higher detection limit, with *LOD*_50_ close to the viral load where someone is substantially infectious (*K*_*m*_), the predicted number of transmissions prevented is lower than for PCR. *LOD*_50_ being close to *K*_*m*_ also makes model results for antigen tests more sensitive to uncertainty in parameters, as demonstrated in Appendix E. Figure 4B shows that daily antigen testing prevents fewer transmissions than daily PCR testing unless the PCR reporting delay is more than a few days (e.g. 1 day for a pathogen with 3 days to peak viral load, and 2 days for a pathogen with 6 days to peak viral load). If antigen testing is done twice daily, Figure 4C shows that it could prevent almost 80% of transmissions; however this result should be interpreted with caution because this model assumes that subsequent test results are uncorrelated when viral load is controlled for (while in reality tests might have shared dependence on factors that might not change for a person in a day). The effectiveness of tests with different parameters can be explored using the app at https://frequent-testing.shinyapps.io/shinyapp.

To estimate the effectiveness of potential mass testing strategies against a broader range of pathogens, we compute *R*_*e*_ while varying both *R*_0_ and time to peak viral load. We find that some strategies might be able to independently control a pathogen. Using the same approach as Figure 4, we find that daily PCR testing with an 8 hour delay could avert 0.96*adherence of transmissions for a pathogen like 1918 influenza (3.5 days to peak viral load, *R*_0_ = 2.5) and 0.98*adherence for a pathogen like wild-type SARS-CoV-2 (5 days to peak viral load, *R*_0_ = 2.5). Here, adherence is defined as the product *γ* · *β* from Equation 7, or equivalently E[Isolation Effectiveness | Tests Regularly] *P(Tests Regularly).

**Figure 5:**
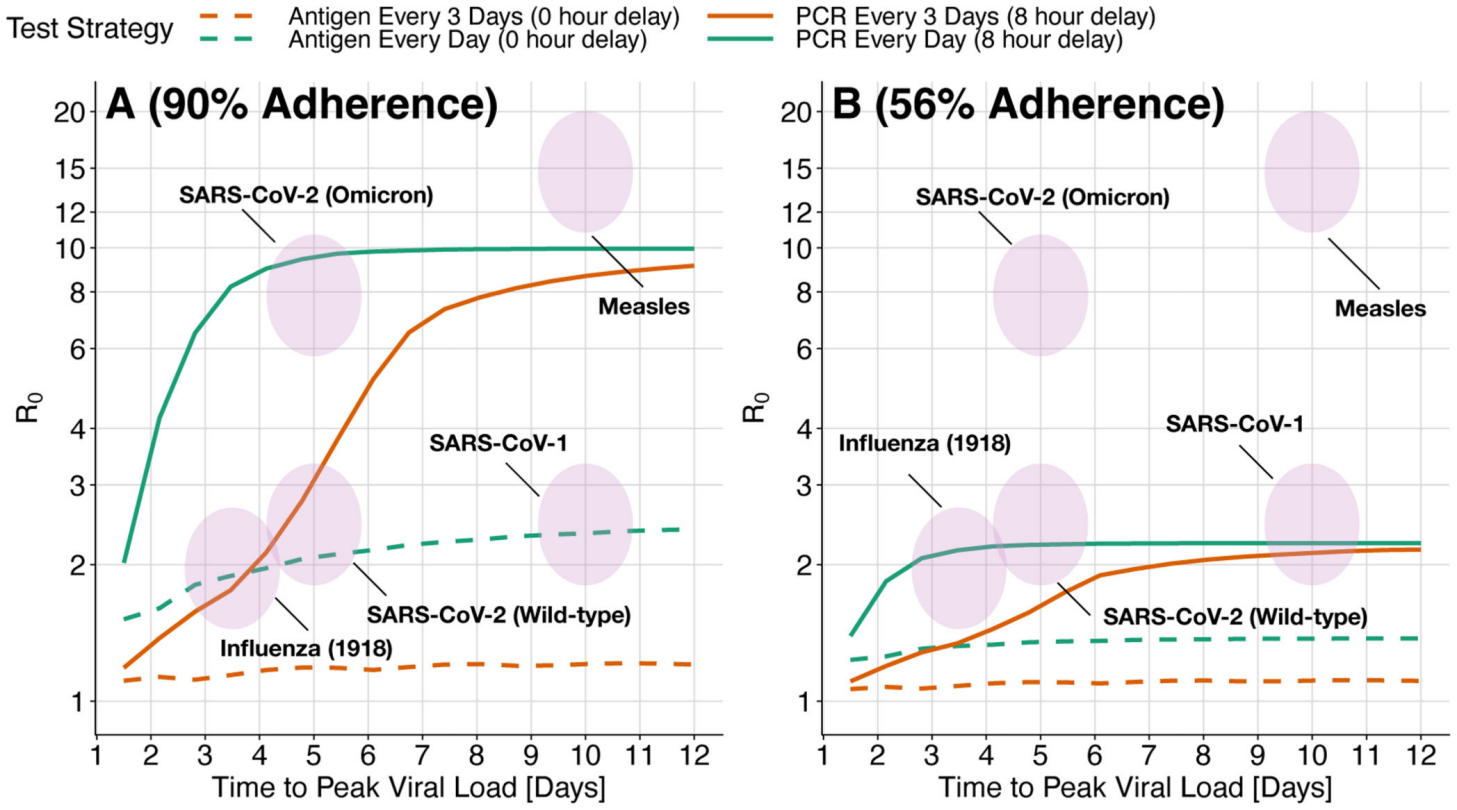
The maximum transmissibility (*R*_0_) that can be controlled by different mass testing strategies depends on the time to reach peak viral load. Viruses in the area under each line can be controlled by that testing strategy alone, in the absence of other measures. High adherence (A not B) and high test frequency (cyan) are required to control a wide variety of challenging viruses. The highest value of *R*_0_ that can be controlled with a testing strategy (computed by increasing peak viral load until *R*_*e*_ in Equation 7 is equal to 1) is shown as a function of time to peak viral load (x-axis), test type (solid vs dashed), and testing interval (orange vs cyan). Shaded ovals indicate the approximate values of *R*_0_ and time to peak viral load for a variety of viruses. Panel A) shows high adherence: 95% adherence to testing and 95% adherence to isolation if positive. The maximum *R*_0_ depends on the product of these two numbers. Panel B shows more moderate 70% adherence to testing followed by 80% isolation adherence if positive. Moderate adherence (B) might be inadequate on its own to control an outbreak of the original wild-type SARS-CoV-2 variant, but makes an important contribution when combined with other measures. As the speed of viral exponential rise and fall (x-axis) is varied, the same *R*_0_ is achieved by adjusting peak viral load. For simplicity, symptom onset does not trigger behaviour modification; in Appendix C we show similar results when symptoms are detected 24 hours before peak viral load and cause a 75% reduction in contacts (while holding *R*_0_ constant and increasing the peak viral load to compensate for fewer contacts).

Mass PCR testing with overall adherence of 90% and an 8 hour reporting delay is sufficient, on its own, to control all of the example pathogens in Figure 5A except for measles (which has a very high *R*_0_). Lines in Figure 5, each representing a different testing policy, indicate the highest controllable value of *R*_0_ for each testing policy, found by increasing the peak viral load, *V*_*p*_, until *R*_*e*_ in Equation 7 is equal to 1. Figure 5A shows very high adherence (95% adherence to testing, and 95% effective isolation), while 5B shows moderately high adherence (70% adherence to testing followed by 80% isolation adherence if positive). While antigen testing in Figure 5 is insufficient on its own to control most pathogens, it can still substantially reduce the number of transmissions that are most difficult to control otherwise: transmissions from cases that have high viral load and are therefore highly contagious even during brief exposures. Using antigen tests with a lower *LOD*_50_ value can also dramatically change the results; this can be explored further at https://frequent-testing.shinyapps.io/shinyapp.

While it is impossible to anticipate parameters for novel pathogens (and indeed, parameters are only partially understood for existing pathogens), the predicted effectiveness of frequent PCR testing is quite robust to changes in parameters. Different pathogens might transmit with a different proportionality factor with respect to viral load, due e.g. to how well the virus binds human cells; this will be reflected in different values of *K*_*m*_. The minimum infectious dose for SARS-CoV-2 is thought to be around 100 particles [33] and the minimum infectious dose for any pathogen has to be *≥* 1. Therefore if transmission occurs via similar mechanistic routes to SARS-CoV-2, it is unlikely that *K*_*m*_ will be more than a factor of 100 lower. In Appendix E, we explore the effect of a 100-fold lower *K*_*m*_ and show that antigen tests fail, on their own, to control any of the pathogens, while frequent PCR testing does only slightly worse (Figure 10). Similarly, the effectiveness of PCR testing is robust to behaviour modifications due to symptoms (Appendix C) and to changes in viral load trajectory shape (Viral Load tab of app: https://frequent-testing.shinyapps.io/shinyapp).

Sustained mass testing was infeasible during much of the COVID-19 pandemic, but there is now time to develop testing technology and infrastructure, so that less socially costly disease control measures are available for future pandemics. Promising newer technologies like multiplexed sequencing are becoming faster and more affordable [34]. Alternatively, more mature technology like saliva-based PCR testing can be scaled up with less technical risk, as discussed in Appendix A. In Appendix A we also discuss feasibility of potential implementations in terms of cost, public acceptability, infrastructure, and timing in a pandemic. We find that there are feasible solutions to deploy a relatively inexpensive mass testing program early in a pandemic, but that the primary logistical barrier is building and maintaining enough testing infrastructure in advance. Once built, uses will likely be found; e.g. at the time of writing, there is an acute need for more H5N1 testing of cows and farm workers in the United States[35].

## 4 Discussion

Our findings illustrate that large-scale, rapid testing can effectively control a respiratory virus pandemic, but that adherence to frequent testing and to isolation is crucial to achieve this impact. A partially adhered-to mass testing strategy could also be used to replace more restrictive interventions such as school or business closures. For the wild-type SARS-CoV-2 variant, assuming 5 days to peak viral load and *R*_0_ = 2.5, overall adherence of 61% would be needed to bring *R*_*e*_ < 1 in the absence of other measures. If work from home, masking, voluntary outdoor socializing etc. succeed in achieving *R*_*e*_ = 1.5, then adherence of 34% would be sufficient in a homogeneous population to further reduce *R*_*e*_ < 1 and achieve control. This modest level of adherence seems achievable, and could avoid the need for more draconian lockdowns. However, in scenarios where the test result delay time is long (e.g. 72 hour) compared to the time to peak viral load, mass testing becomes substantially less useful, bringing down from *R*_0_ = 2.5 to *R*_*e*_ = 2.3 and 2.4 in these two examples of 61% and 34% adherence. Contact tracing will magnify the benefits of mass testing [36], and with long test delays, the indirect benefits of testing on contact quarantine become more important than the direct effects on case isolation.

Our results predict that mass testing can effectively reduce transmission across a wide range of pathogen parameters - encompassing most known respiratory pathogens. The main exception is extremely high *R*_0_ viruses like measles, which would require very high testing and isolation adherence. While the specific characteristics of future pathogens cannot be known in advance, our results suggest that rapid deployment of mass testing would likely be an effective containment strategy across most plausible parameter combinations.

Our model assumes a well-mixed population, but we note that if there is a connected group of people that are unable or unwilling to adhere to testing or isolation, then the epidemic could escape control within that sub-network. Conversely, as seen for SARS-CoV-2 in some universities and film studios, smaller groups of people might adopt frequent testing to control transmission within their sub-network, in scenarios where there isn’t broader public support for disease elimination. The smallest, simplest example of this is testing the caregiver for someone at elevated risk of severe disease, reducing the caregiver’s transmission probability by the amount shown in Figure 4.

A scenario where massive but non-universal testing capacity could be useful is to avoid school closures by frequently testing students and staff. School closures in the US were estimated to have cost $6 trillion USD during COVID-19 pandemic, but some schools avoided closures by testing frequently. If testing were done frequently enough and a negative test were a prerequisite for attendance, then the probability of an infected contact at school transmitting the disease would be 10-20 times lower than otherwise (see Figure 4 for the 90-95% fraction reduction in transmissions, depending on the virus). Unless closing schools reduces childrens’ contacts by a factor of at least 10-20, effective testing at school could actually be safer than closing schools and not testing. This might be a good use of testing capacity that, while massive, is nevertheless insufficient on its own to control a pandemic - potentially not avoiding all social distancing measures, but avoiding school closures. While the school setting might have above the usual *R*_0_, it is also conducive to the enforcement of testing and isolation policies.

In a scenario where a pathogen causes sufficiently severe disease such that most people adopt social distancing, essential workers might choose not to work because of fear of infection. Frequent testing of essential workers and the people they interact with could dramatically improve their safety (in addition to other important safety measures like effective PPE [37]), and reduce the probability of infecting workers’ family, which could increase the number willing to continue working [38].

Frequent mass testing of asymptomatic individuals inevitably risks large numbers of false positive tests leading to isolation both of individuals who are not infected, and of individuals who were infected but are no longer infectious. However, even with wide-scale testing, the total number incorrectly isolated is manageable and fairly small, despite an expected reduction in positive predictive value as true positive individuals are removed from the population and thus prevalence in the remaining tested population goes down [39]. The duration of unnecessary isolation could be shortened by ending isolation earlier given a series of negative results, and/or by using a viral load threshold during the downward trajectory, rather than isolating until completely negative.

This work focuses on population-wide asymptomatic testing, but given limited testing capacity, it is most efficient to prioritize symptomatic testing before asymptomatic testing [40]. For simplicity, we did not model people seeking additional testing because of symptoms, although we did model behavior modification due to symptoms even in the absence of a positive test. In a related work, we use a branching model to estimate the benefit of adding symptomatic testing and contact tracing to a mass testing policy, finding that in some scenarios adding contact tracing could allow the mass testing frequency to be halved with the same reduction in transmissions [36].

We made the simplifying assumption of transmission risk being independent of location and time, but testing policies could be made more efficient by focusing on higher risk settings. For example, collecting samples from people so that their test results are returned right before attending an event would prevent even more transmissions. A location-based strategy could target more frequent testing to regions with an active outbreak, and less frequent surveillance testing for new outbreaks in previously clear regions, requiring fewer tests in total as a country approaches elimination.

The COVID-19 pandemic caused an estimated $16 trillion USD in harm in the USA [41], and efforts to contain it were also very costly (e.g. $2.5 trillion USD in lost future productivity from four months of school closure in the USA [42]). Another pandemic at least as severe as COVID-19 is likely in the next few decades, and many countries are not prepared. We show that with sufficient testing capacity and good adherence, mass testing could be effectively used to mitigate transmission and avoid the much more costly interventions and harms of future pandemics or reactions to future pandemics. This motivates substantial effort to design a testing system with high enough throughput, and to build it before the next pandemic.

## Supplementary Material

Appendices

## Figures and Tables

**Table 1:** Parameters and variables for modelling viral load, test sensitivity, and expected transmissions.

Model Component	Description

*V* (*t*; *V*_0_*, V_p_, τ_p_, τ_r_*)	Viral load at time *t* since infection. Dependence on other param- eters is described in Equation 1 and shown in Figure 1A.
*V*_0_	Viral load at the time of infection: *V*_0_ = 3 · 10^−3^ based on one viral particle within ≈ 300ml of respiratory fluid [26].
*V_p_*	Peak viral load. Adjusted to attain a specified value of *R*_0_.
*τ_p_*	Time from infection to peak viral load: 1.5 to 12 days.
*τ_r_*	Time from *V_p_* to *V*_0_. Symmetry assumption (*τ_r_* = *τ_p_*) can be relaxed in the app.
*S*(*v*; *S_max_, k, LOD*_50_)	Test sensitivity (probability that a positive sample is detected as positive) as a function of viral load, *v*. Dependence on other parameters described in Equation 2 and shown in Figure 1B.
*S_max_*	Maximum test sensitivity: 99.5% for PCR, 90% for antigen tests.
*k*	Controls width of intermediate test sensitivity region: *k* = 6 for PCR and *k* = 1.3 for antigen tests.
*LOD*_50_	Viral load at which test sensitivity reaches *S_max_/*2: 10^2^ copies/ml for PCR and 10^5.4^ for antigen tests.
*T* (*v*; *N_C_, h, K_m_, θ*)	Expected transmissions per day as a function of viral load, *v*. Dependence on other parameters described by Equation 3 and shown in Figure 1C.
*N_C_*	Average number of close contacts per day: *N_C_* = 13 based on [28]. Appendix C explores time-dependent reduction in contacts caused by symptoms.
*h*	Controls width of sigmoid for transmission risk vs. viral load: *h* = 0.51 based on [29].
*K_m_*	Midpoint of infectiousness: *K_m_* = 8.9 · 10^6^ copies/ml based on [29].
*θ*	Increased from 0.2 in Ke et al. [29] to 0.3, corresponding to a maximum per-contact transmission probability of 26%.
*σ*(*δ, ρ*)	The fraction of transmissions that would be prevented (relative to *R*_0_) if the entire population tested every *ρ* days and received test results after *δ* delay.
*δ*	Time delay before receiving a test result. 0 for antigen tests and 2+ hours for PCR.
*ρ*	Time between tests.
*γ*	Fraction of the population that adheres to regular testing.
*β*	Fraction reduction in transmissions after a person who tests regularly learns they are positive.
*R*_0_	Expected transmissions per infected person with no population immunity or disease-control measures.
*R_e_*	Expected transmissions per infected person given interventions, with no population immunity.
