# Supplementary material for: Enhanced testing can substantially improve defence against several types of respiratory virus pandemic
Source: Epidemics. Author manuscript; Available in PMC 2025 Jul 30. (PMC7617967; doi:10.1016/j.epidem.2024.100812)
Supplement: Appendices [file EMS207241-supplement-Appendices.pdf]

Appendices

A Implementation Feasibility

For a mass testing strategy to be effective, there are several things that must go right: (1) tests must be available early in a pandemic; (2) there must be scalable logistics for fast transportation of tests/samples; (3) there must be sufficient public support for the strategy; (4) this support must be translated into sufficient adherence to testing and effective isolation; (5) there must be enough test equipment and skilled technicians; (6) the variable cost per test must be affordable. While these challenges are significant, the massive costs of either social distancing or unchecked disease, and the difficulties of effective contact tracing, motivate a thorough investigation of mass testing as a potential solution, either as an alternative to distancing and contact tracing, or as an adjunct.

In this Appendix we focus on PCR testing of saliva samples because it is a mature technology that has been proven at scale and can be functional within weeks of detecting a pandemic. Rapid antigen testing is another option that has been proven at scale, but for it to be useful in future pandemics, development, manufacturing, and deployment would have to be much faster than they were during the COVID-19 pandemic. Specifically, rapid antigen tests became available almost a year after detecting the COVID-19 pandemic [43]. There are also several other promising technologies, e.g. based on multiplexed next-generation sequencing [34, 44], LAMP [45], CRISPR [46], or even particle imaging [47]. The aim of this Appendix is to demonstrate one relatively low-risk strategy for scaling up, but not to argue that it is the best or only possible solution for mass testing. In the ideal case, redundant technological approaches and infrastructure could improve robustness to unanticipated challenges.

A.1 Time to Deploy

PCR can be ready within weeks of detecting a pathogen; e.g., Corman et al. published a protocol for SARS-CoV-2 on January 23, 2020, 13 days after the pathogen sequence was first published [48]. The main decision that needs to be made is which amplicon targets to use to selectively amplify RNA or DNA from the pathogen of interest (e.g. distinguishing SARS-CoV-2 from other coronaviruses) [49]. After a protocol is chosen, oligonucleotides for the target amplicons can be synthesized with a high-throughput solid phase process [50] and distributed to testing labs. Redundancy in protocol design and test kit manufacturing would be prudent to avoid delays like the ones experienced by the USA in early 2020 [51].

A.2 Logistics: Sample Collection and Transportation

Cost-effective deployment at scale could be achieved by borrowing approaches from home delivery services, a market that has recently expanded and successfully overcome many logistical challenges. As an example, both unused sample collection kits and self-collected saliva samples could be left at unstaffed booths (potentially with security cameras to deter misuse). This could be done cheaply by providing a tray of empty tubes from which users detach a QR code, collect and seal a saliva sample, and place the tube in a box. Rideshare or delivery companies could be hired on demand to frequently (i.e. ideally with less than 4 hours wait time to achieve a total turnaround less than 8 hours) pick up the boxes of samples at a low average cost per sample (e.g. if 50 samples were transported by a $30 USD Uber ride). In low or middle income countries (LMICs) the available budget would likely be lower, but the cost of labour would also be lower. The right choices for logistics depend on the setting, but using self-collected samples with batched delivery through existing consumer delivery services or similar could likely keep the cost of logistics below $1 per sample in high income countries and considerably lower in LMICs.

A.3 Public Support

Mass testing depends on public support, which could potentially be improved by making the overall approach simple and non-invasive. For example, several schools used saliva or gargle based testing because it avoids the discomfort of nasopharyngeal swabs [52] and samples are easily self-collected without specialized supplies. Saliva tests sometimes had higher sensitivity early in infection [53], although the cheap, low-volume SalivaDirect test had slightly worse sensitivity [54]. Using easily accessible locations where tests can be completed with a few minutes could also improve the overall experience. Because public support is so important for mass testing to be successful, it is likely worthwhile to make significant trade-offs in test sensitivity, delay time, or cost if necessary. However, if there remains insufficient public support for mass testing of the entire population, testing could be focused on schools and senior living facilities.

A.4 Testing and Isolation Adherence

The achievable level of testing and isolation adherence depends on public sentiment, which likely depends strongly on the severity of the pandemic. In milder pandemics, adherence might depend on voluntary participation, as enforcement might be too extreme an infringement on civil liberties. However, in a very deadly pandemic without viable alternative control strategies, there might be support for more strictly enforcing adherence. This might be easier if the verification process is smooth, and if those who do not wish to adhere have some viable (albeit restrictive) alternative option to partially quarantine rather than test. E.g., proof of a recent negative test could be required for entry into public spaces where there is substantial risk of transmission (similar to testing requirements for flights or some schools or workplaces for SARS-CoV-2).

Data on the effectiveness of home isolation is limited [55], but isolation in dedicated facilities is likely to be highly effective. Even quarantine in poorly chosen facilities that mixed air between the infected and uninfected was shown to be at least 99.4% effective at preventing transmissions of SARS-CoV-2 from infected cases in Australia [56]. Isolation could be incentivized by fully replacing lost wages (the COVID-19 pandemic typically saw partial wage replacement at best [57]), and providing generous support (e.g. accommodation, food, childcare, medical care) [55]. Successful local control of a pandemic corresponds to fewer than 1/1000 people infected at any time (and hopefully far fewer), meaning that spending double each person's salary to support their isolation would cost less than 2/1000 of GDP.

A.5 Testing Capacity: Equipment and Skilled Labour

Test capacity depends on equipment and skilled labour. Capacity was a significant barrier to performing mass testing during the COVID-19 response when there was little time to manufacture, let alone design, new equipment. Sample pooling was sometimes used to test more people using a fixed amount of available equipment, at the expense of decreased sensitivity, increased complexity, and many re-tests when prevalence is high [58].

A simple way to build PCR capacity in preparation for future pandemics is to manufacture more conventional PCR equipment, to be combined with sample pooling. Minhas et al's description of a national lab in Pune, India [59] can be used as a basis to estimate the upfront investment and number of trained staff needed for this strategy, although we note that locations with high labour costs would probably employ fewer people while using more expensive equipment. Their peak capacity was 1800 samples per day, they employed around 100 people, and the cost of their diagnostic equipment was 15,592,420 INR, which is roughly equivalent to $188,000 USD. The same lab could process samples that were pooled 10x at time of collection (having all 10 individuals home isolate until individually retested), thus processing roughly 18,000 samples per day. 100 people employed in a lab per 20,000 sampled is 1/200 of the total population; this would be highly costly to staff in preparation. As a comparison, 1/2000 people are on 'standby' as firefighters in the UK [60]. Rapidly recruiting skilled technicians when a pandemic is detected is another option, but this would be difficult to do in 1-2

months and only about 1/1000 people are currently employed in the medical diagnostics industry in the US [61].

Alternatively, there has been recent progress on highly automated workflows for PCR diagnostics [62, 63, 64] that require fewer lab technicians. One example is endpoint PCR [65], which uses a waterbath instead of the more common piezoelectric system, for higher-throughput thermal cycling at lower cost. A large British diagnostics company, LGC Group, claims to be able to test 150,000 samples per day [66] with two technicians per shift and an upfront cost of $902,000 USD for the equipment [personal communication]. We note however that a real-world deployment of this technology only reached 65,000 samples/day with an infrastructure cost of $186M USD [67]. The reasons for such a high cost include construction of a 220,000 square foot facility, while the low output likely reflects reliability issues with early-stage technology [68].

One strategy for building and maintaining high-throughput PCR for the purpose of pandemic readiness, within a developed country, is to make research institutions eligible for targeted grants. These grants would provide standardized ultra-high-throughput PCR equipment, which the grantee would be free to put to innovative research use between emergencies. In exchange, grantees would guarantee a mobilizable workforce able to use the equipment for mass testing, as assessed through a system of occasional drills. Once established, it might be found that mass testing is useful more often than anticipated, e.g. at the time of writing, there is an acute need for more H5N1 testing of cows and farm workers in the United States [35]. Such a scheme would have the benefit of standardizing the equipment, once an appropriate prototype is available. The current LGC technology would first need clearer proof of concept before being chosen, and it is possible that more R&D is first required to build an adequate system.

A.6 Variable Cost per Test

Even when there is sufficient equipment and trained personnel, there is a per-test cost that determines how much countries can afford to test in a pandemic. This cost depends on the price of consumables and the price of logistics (discussed in Section A.2), and was fairly high in most countries during the COVID-19 pandemic (e.g. $24 - $55 USD per test in the UK [69]). Pooled testing substantially reduced the cost of PCR testing during the COVID-19 pandemic by using less consumables per sample (e.g. Mirimus Inc. tested students in New York for $10/sample [70], and China tested millions of people for $1.50/sample [58]). SalivaDirect demonstrated that low prices could be achieved by using a small volume of the cheapest available reagents, reducing the cost of consumables to $1.21/sample with an extraction free protocol [54]. In Section A.5 we argued that the cost of logistics in high income countries could be less than $1 per sample, so with consumables included the cost per test could be around $2. For wealthier countries, a cost of $2/person/day is very affordable if it averts most of the harm of the pandemic, and for LMICs, the cost could potentially be further reduced with sample pooling and when considering the lower cost of delivery. For the USA, the cost of testing every person daily for a year would be about $240 billion, which is almost 10 times smaller than the $2.2 trillion spent on the CARES Act [71] in response to the COVID-19 pandemic.

B Impact of Heterogeneity in Transmissibility and Time to Peak Viral Load

We modeled each pathogen as having a fixed expected number of transmissions (R_0) and time to reach peak viral load (τ_p) for every infected person. In reality, viral load trajectories will be different for every person who is infected, which could be more accurately modelled as drawing R_0 and τ_p from a distribution. By only computing R_e at the average parameter values instead of integrating over the entire parameter distribution we might introduce some error. I.e., we made the approximation that $E[R_e(\tau_p, R_0)] \approx R_e(E[\tau_p], E[R_0])$, where $[\tau_p, R_0]$ is a random vector.

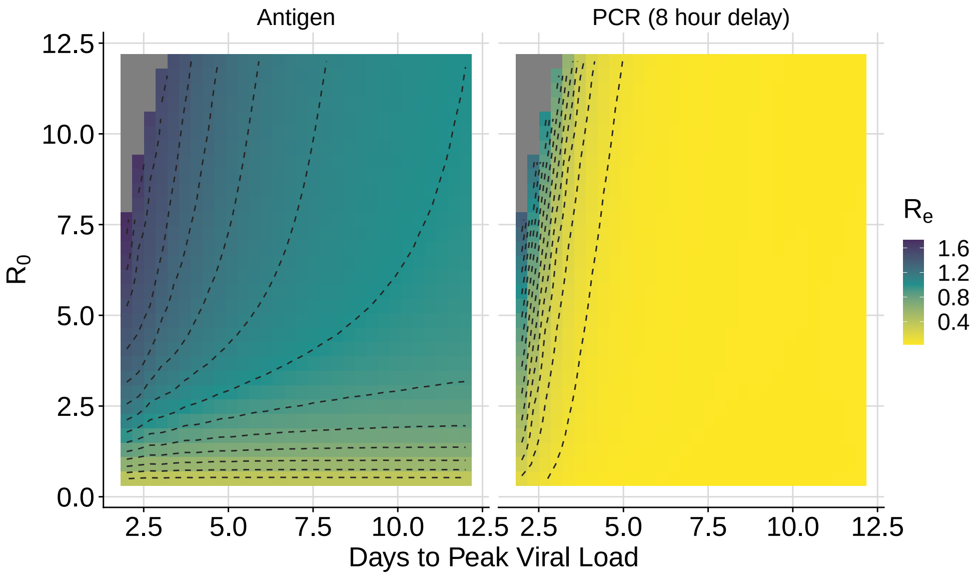

Figure 6: R_e vs. R_0 and τ_p for daily antigen testing (with immediate results) and PCR testing (with an 8 hour result delay).

If $R_e(\tau_p, R_0)$ is a linear function, then this approximation is exact. If $R_e(\tau_p, R_0)$ is well approximated by a linear function in the neighborhood occupied by the parameter distribution for a pathogen, then the error introduced by this approximation is therefore small. Figure 6 shows how R_e depends on R_0 and τ_p for PCR tests and antigen tests (assuming perfect adherence - lower adherence can be computed with a linear transformation of the displayed function). Approximately linear regions in the function are shown as either not having any contour lines (flat) or having consistent spacing between straight contour lines (increasing linearly in the direction of a constant vector). For both types of tests the function is nonlinear when $\tau_p < 3$ days, and for antigen tests the function becomes nonlinear with lower values of R_0. If the $[\tau_p, R_0]$ distribution for a pathogen does not have much mass in these non-linear regions, then the point approximation does not cause significant error.

C Sensitivity to Symptom-Based Behaviour Modification

Modified assumptions regarding timing of symptoms and behaviour change due to symptoms do not substantially change the estimated effectiveness of mass testing. Figure 7 is generated using the same parameters as Figure 3, except symptoms occur 24 hours before peak viral load instead of at the time of peak viral load, and contacts are reduced 75% instead of 50% after symptoms. Because R_0 is held at the same value of 2.75, the peak viral load for the scenario with earlier symptoms is higher. The predicted impact of testing in these two scenarios is almost identical, except with earlier symptoms, slightly more frequent testing is needed. This is mainly because the increase in peak viral load shifts more transmissions earlier in the infection, which requires more frequent testing to detect in time. Similarly, Figure 8 shows a modified version of Figure 5 in the scenario where symptoms are detected 24 hours before peak viral load, and cause a 75% reduction in transmissions. For a fixed R_0, the addition of symptoms changes the shape of the infectiousness profile. Daily testing performs slightly worse in this scenario because more transmissions occur earlier in infection. With testing every 3 days for fast viruses, the increase in test sensitivity from a greater viral load is a more important effect, causing the testing effectiveness to increase slightly.

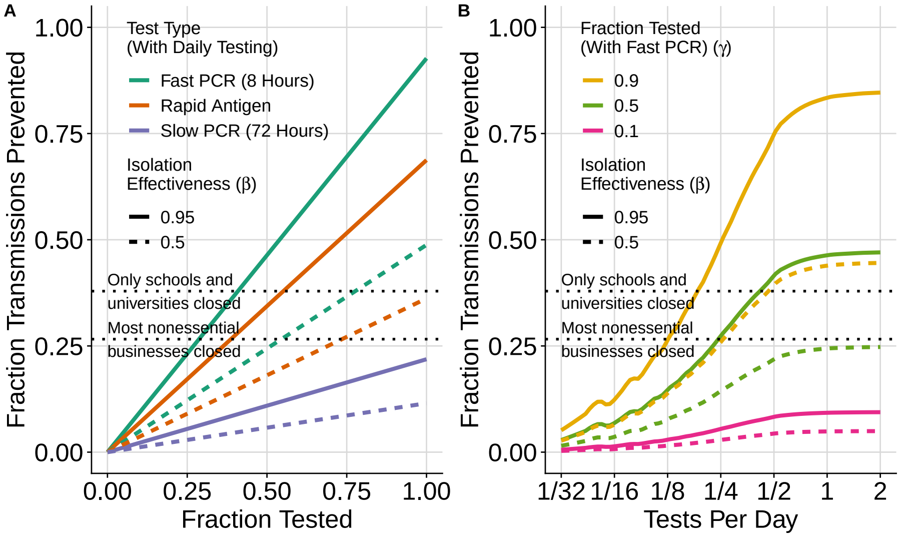

Figure 7: **Fraction of (ancestral variant) SARS-CoV-2 transmissions prevented when symptoms occur 1 day before peak viral load, and contacts are reduced by 75% after symptoms.** Compare to symptoms 0 days before peak viral load and a 50% reduction in contacts in Figure 3). Because R_0 is kept at 2.75, the additional behaviour modification from symptoms causes the computed peak viral load to increase slightly, causing transmissions to shift earlier in infection. Except for a slight reduction in the effectiveness of slow PCR tests, the modified symptom parameters do not substantially change Figure 3.

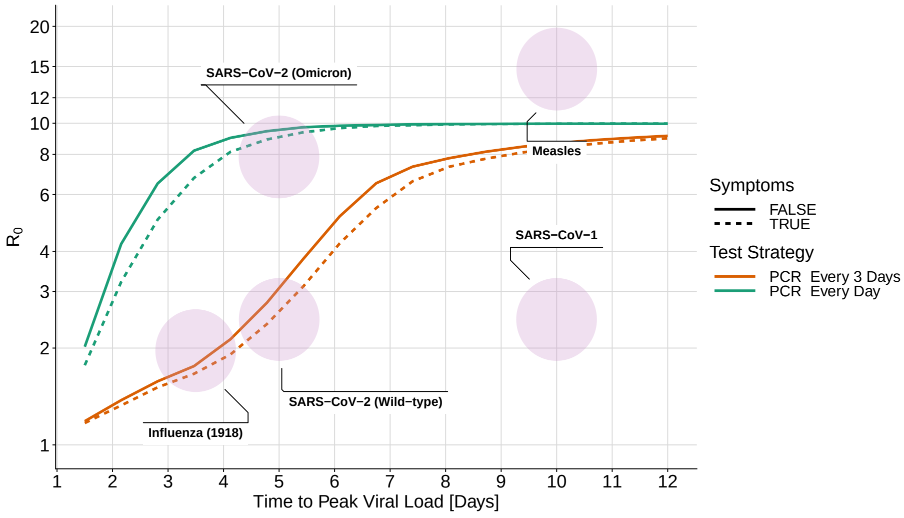

Figure 8: **Effectiveness of mass testing with and without behaviour modification due to symptoms.** As in Figure 5, the solid lines do not include symptoms. The dashed lines are computed with symptoms 24 hours before peak viral load and a 75% reduction in contacts after symptoms. For a fixed value of R_0, the addition of symptoms causes the computed viral load to increase and transmissions to shift earlier in infection. Earlier transmissions are generally more difficult to control, so the effectiveness of mass testing decreases slightly because of this.

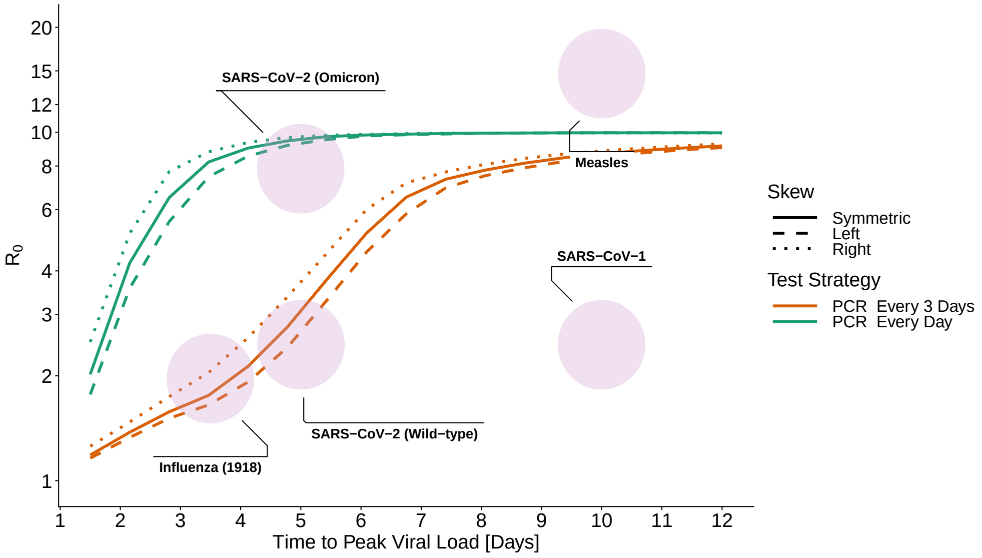

Figure 9: Maximum R_0 controllable by each testing strategy (as in Figure 5), with different viral load trajectory skews. Symmetric skew ($\tau_p = \tau_r$) as in the main text, left skew ($\tau_p = 2\tau_r$), and right skew ($2\tau_p = \tau_r$).

D Sensitivity to Skew of Viral Load Trajectory

Figure 9 shows the maximum controllable R_0 with different viral load trajectory skews. With right skew trajectories, control is slightly more effective, and with left skew trajectories control is slightly less effective. The overall results are similar despite the significant change in trajectory shapes.

E Sensitivity to Infectiousness Midpoint

To test the sensitivity of our results to the infectiousness function in Equation 3, we reduce the viral load at which half of peak infectiousness has been reached from $K_m = 8.9 \cdot 10^6$ to $K_m = 8.9 \cdot 10^4$. With a 100 times smaller value of K_m, the viral load needed to transmit is substantially lower, while the viral load needed to detect infection stays the same. In Figure 10 we see that with the reduced value of K_m, frequent PCR testing does slightly worse than in Figure 5, while antigen testing fails to control any of the pathogens. This is because in this extreme scenario, PCR tests are still able to detect infections before people become substantially infectious, while the antigen tests fail to detect infected people even at their highest viral load.

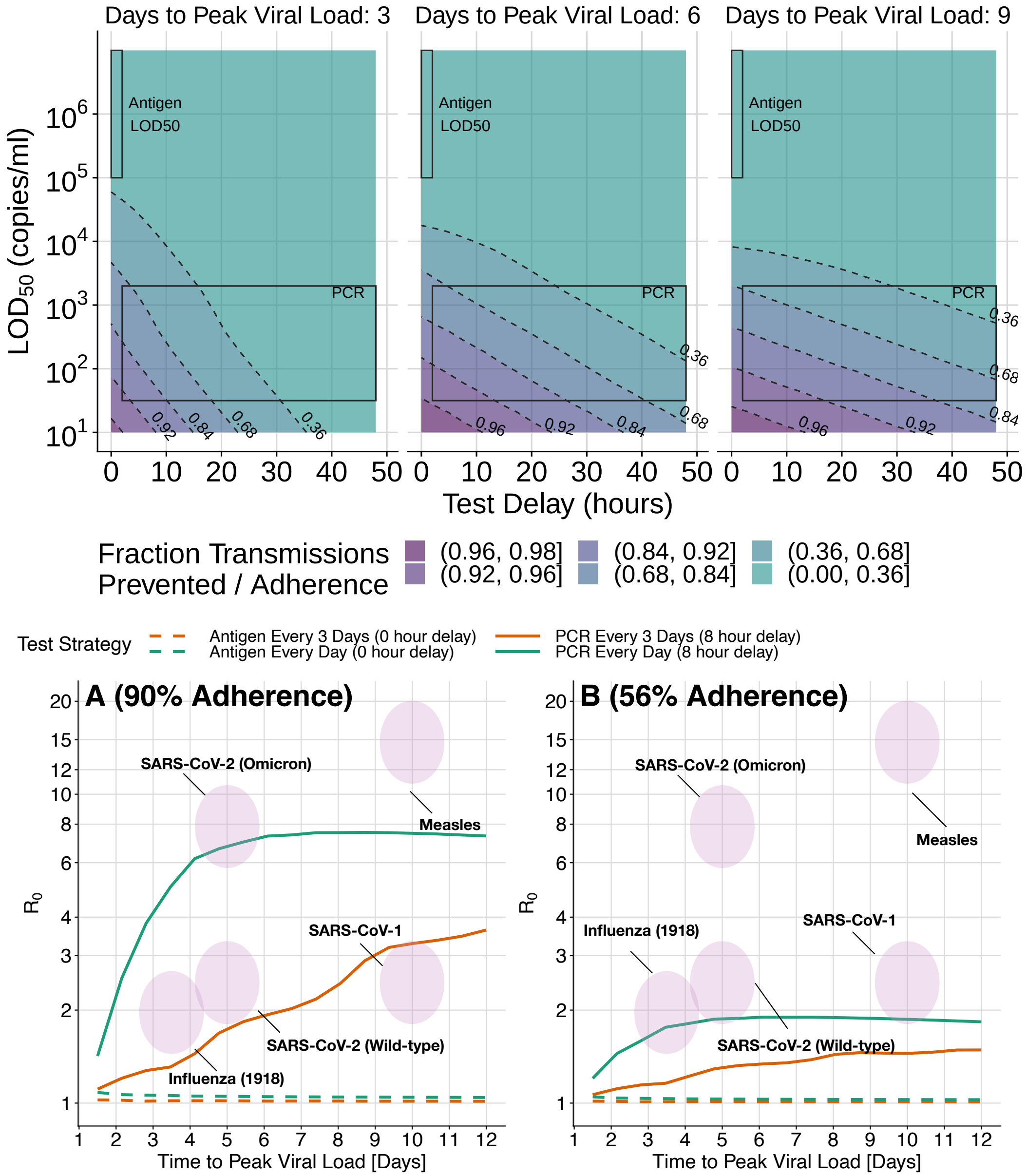

Figure 10: Figures 4 and 5, are recomputed with the viral load midpoint for infectiousness reduced from $K_m = 8.9 \cdot 10^6$ to $K_m = 8.9 \cdot 10^4$. In the upper 3 panels we see that for PCR tests, the number of transmissions prevented is slightly lower than in Figure 4, and for antigen tests less than 30% of the transmissions are prevented. In the bottom two panels, PCR tests are able to control a similar range of epidemics as in Figure 5 (with slightly reduced effectiveness) and antigen tests are unable to substantially control any outbreaks.
